# Predictability of state-level flood damage in the conterminous United States: the role of hazard, exposure and vulnerability

**DOI:** 10.1038/s41598-017-05773-4

**Published:** 2017-07-13

**Authors:** Qianqian Zhou, Guoyong Leng, Leyang Feng

**Affiliations:** 1 School of Civil and Transportation Engineering, Guangdong University of Technology, Waihuan Xi Road, Guangzhou, 510006 China; 2 Joint Global Change Research Institute, Pacific Northwest National Laboratory, College Park, MD USA

## Abstract

Understanding historical changes in flood damage and the underlying mechanisms is critical for predicting future changes for better adaptations. In this study, a detailed assessment of flood damage for 1950–1999 is conducted at the state level in the conterminous United States (CONUS). Geospatial datasets on possible influencing factors are then developed by synthesizing natural hazards, population, wealth, cropland and urban area to explore the relations with flood damage. A considerable increase in flood damage in CONUS is recorded for the study period which is well correlated with hazards. Comparably, runoff indexed hazards simulated by the Variable Infiltration Capacity (VIC) model can explain a larger portion of flood damage variations than precipitation in 84% of the states. Cropland is identified as an important factor contributing to increased flood damage in central US while urbanland exhibits positive and negative relations with total flood damage and damage per unit wealth in 20 and 16 states, respectively. Overall, flood damage in 34 out of 48 investigated states can be predicted at the 90% confidence level. In extreme cases, ~76% of flood damage variations can be explained in some states, highlighting the potential of future flood damage prediction based on climate change and socioeconomic scenarios.

## Introduction

A growing body of research has investigated flood damage arising from hydro-climatic extremes due to its devastating impacts on society and environment, in particular in the context of climate change and socioeconomic development^1–7^. In the United States, flood damage ranks as the top weather-caused loss and is still on the rise with increasing extreme weather and socioeconomic growth^5, 7–13^. Over the second half of the 20th century, annual losses by US floods have almost tripled from $1.7 billion/year in the 1950s to $5 billion/year in the 1990s (all in 1995 dollars)^14^. Against this background, a better understanding of the drivers of flood damage is crucial for improving our capabilities in predicting flood losses for better adaptations^4, 15, 16^.

Changes in flood damage can be attributed to not only the changes in frequency and magnitude of natural hazards (e.g., precipitation and runoff extremes), but also the level of exposure (the population and economic assets located in flood hazardous areas) and vulnerability (the susceptibility of the exposed elements to hazards) in flood prone areas^17–22^. Empirical approach has been well adopted in previous studies for linking flood damage to a set of hazard, exposure and vulnerability indicators^23, 24^. This allows prediction of future flood damage based on historical relations fed with scenarios of future flood hazard, exposure and vulnerability conditions^25–27^. To serve different purposes of damage estimation, the selected indicators and the temporal and spatial scales vary largely in previous studies. Hazards are commonly described by climate variability, such as extreme precipitations. Flood hazards are then related to exposure and vulnerability indicators which account for social and economic conditions, ranging from highly aggregated factors (e.g., GDP, population) to localized ones (e.g., building type and value, defense facilities)^16, 20, 28, 29^. During the past decades, a stronger signal of change in the frequency rather than in the magnitude of flood events has been reported^11–13, 30^, which is well correlated with the increase in flood damage in the United States^31, 32^. *Pielke and Downton* ^14^ showed that the 2-day heavy rainfall events and the number of wet days have significant correlations with observed flood damage at the national level, which are further regulated by socioeconomic factors. However, *Changnon et al*.^9^ argued that most of the upward trend of flood damage is induced by socioeconomic development resulting in larger exposure and vulnerability to flood hazards. *Choi and Fisher* ^33^ stated that growth in reported flood damage from weather-related disasters is mainly caused by three socioeconomic factors: inflation, growth of population and per capita wealth.

Built upon previous studies, this paper advances our understanding on US flood damage through: (1) assessing the spatial pattern of observed trends in flood damage at the state level. Previous studies on US flood damage assessments were typically carried out at the national or regional level^9, 14^, which ignored the spatial heterogeneities of hazard, exposure and vulnerability indictors. In fact, trends in flood damage and the influencing factors may differ greatly in individual states under various weather, topographical, demographic and economic conditions; (2) considering runoff as one of the physical hazard indicators to explain flood damage variations. Historical flood damage is commonly linked to precipitation related hazards in previous studies^5, 14, 31, 34^, without considering runoff related factors. Runoff can reflect the combined influence of climatic (e.g., precipitation) and land surface conditions (e.g., topography, soil and land cover), which would theoretically better explain flood damage variations than precipitation; (3) investigating the role of exposure as well as vulnerability in regulating flood damage response to hazards. Gross domestic product (GDP) and Population (POP) are the most commonly used indicators to describe exposure and vulnerability of a region^14, 33, 35^. However, it gives limited information on what (e.g., changes in land use or asset values) has driven the increasing flood damage; (4) exploring the potential of flood damage prediction based on revealed empirical relations for each state.

## Results

### Spatial and temporal changes in flood damage and socioeconomic conditions

**Figure 1:**
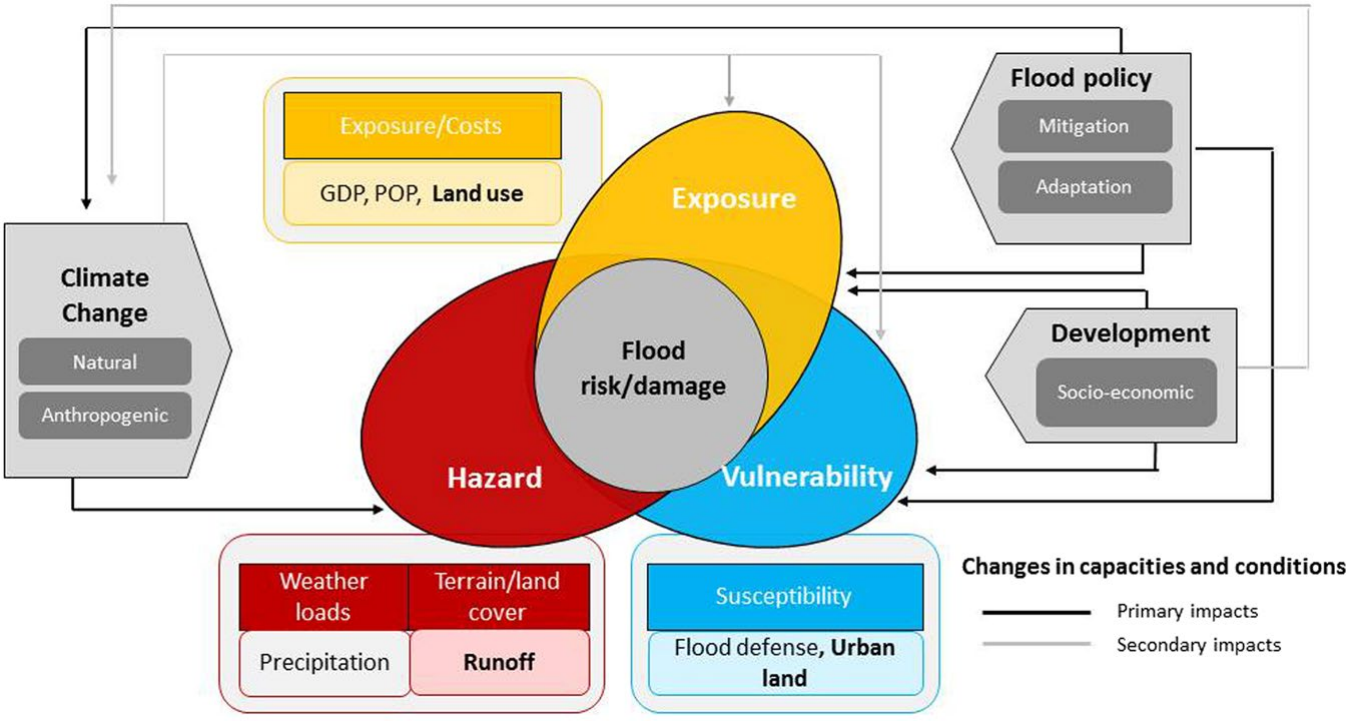
Conceptual framework for analyzing flood damage variations. Figure was created using Adobe Illustrator CS6 software (http://www.adobe.com/products/illustrator.html).

**Figure 2:**
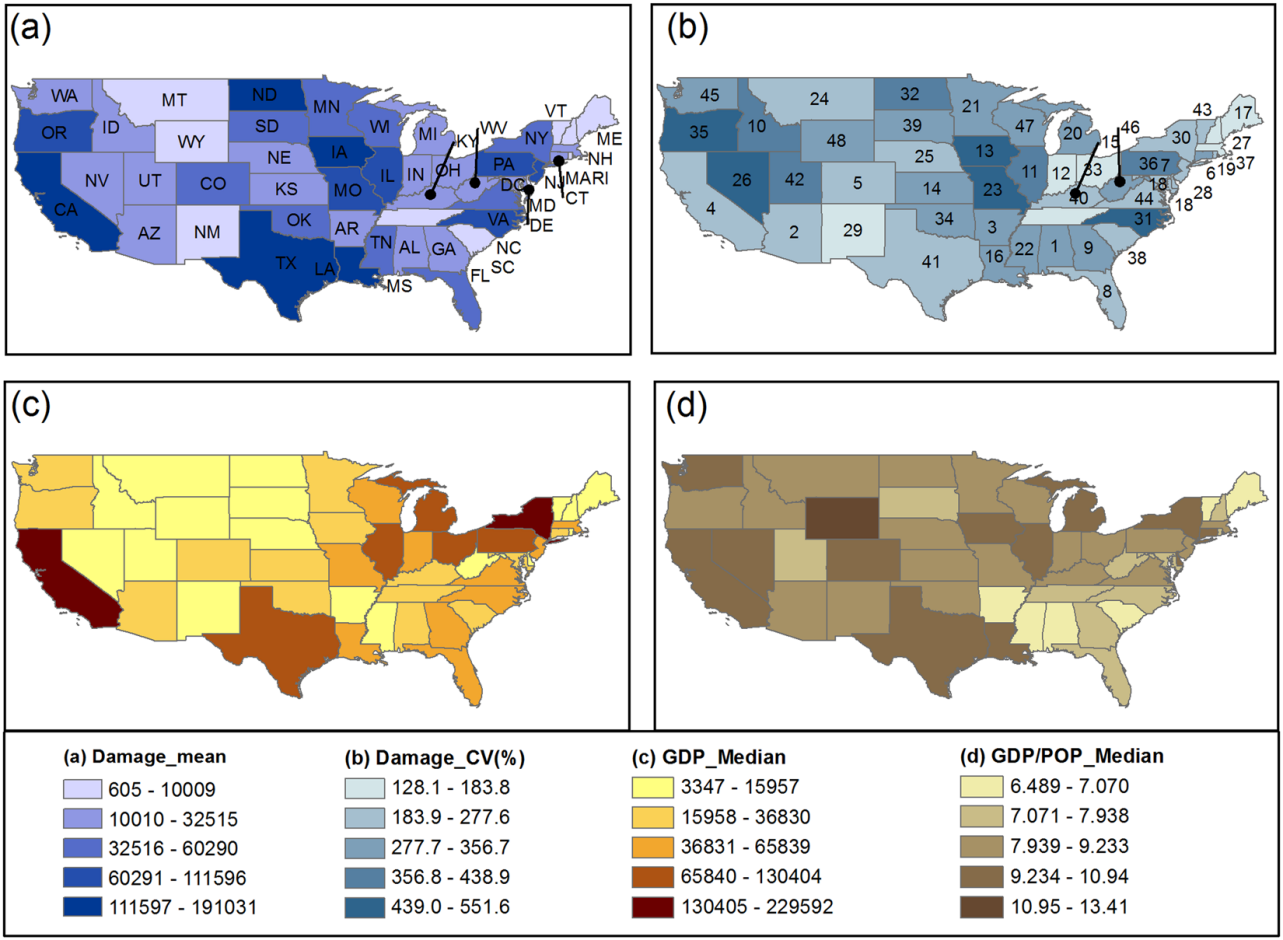
Spatial distribution of state-level (**a**) mean annual flood damage (in thousands of current dollars), (**b**) the standard deviation of flood damage normalized by the mean (i.e., coefficient of variation, CV), (**c**) median GDP (in millions of current dollars) and (**d**) median GDP/median POP (in thousands) for the period of 1955–99. Figure was created using software ArcGIS 10.1 (http://www.esri.com/software/arcgis).

As illustrated in Fig. 1, flood damage is a combined product of flood hazard, exposure and vulnerability. Here, we first show the spatial and temporal patterns of the indicators for flood damage, hazard, exposure and vulnerability before examining the mechanisms behind flood damage. A distinct spatial pattern is observed for historical flood damage across CONUS with highest damage occurring in the state of Louisiana, Iowa, California, North Dakota and Texas (Fig. 2a). Among the five states, large inter-annual variability of flood damage is found in Louisianan and North Dakota (Fig. 2b). For the country as a whole, total flood damage during 1955–99 is about 85 billion dollars with mean annual damage up to 1.89 billion. The spatial distributions of median annual GDP and the ratio to POP of each state are shown in Fig. 2 (c) and (d), respectively. It is not surprising to find that the states experiencing highest flood damage, such as California, Texas, Illinois and Pennsylvania, are generally characterized with higher levels of wealth as indicated by GDP. However, in the state of New York, Ohio and Michigan, despite their high GDP levels, flood damage during the historical period is relatively low, possibly due to the low level of hazard occurrence/magnitudes (Supplementary Figure S1), which are explored in the following analysis. The unit level of wealth (i.e., per capital GDP) is consistent with the gross wealth in the state of California, Illinois, New York and Texas, but with highest value in Wyoming and Nevada given their small population.

**Figure 3:**
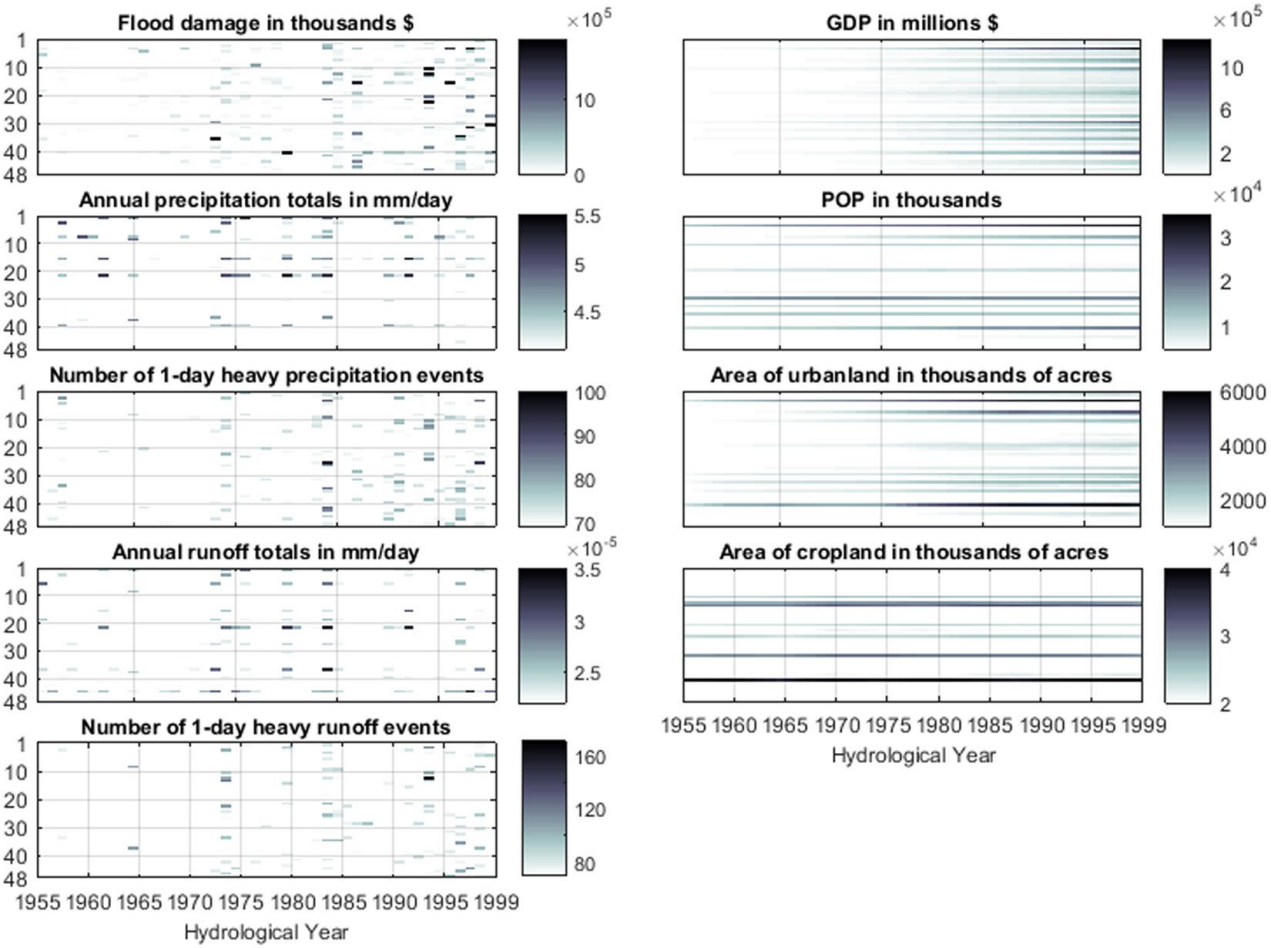
Temporal changes in flood damage and the corresponding hazard and social-economic factors in 48 investigated states during 1955–99. The y-axis indicate the state ID with the location shown in Fig. 2b. Figure was created using software MATLAB 2015a (http://www.mathworks.com/).

Temporally, flood damage shows an increasing tendency in the study period, and more than two thirds of major damaging events (highlighted in bone color) occurred over the last 15 years (Fig. 3). In particular, the hydrological year 1993 is the most damaging and devastating year in the country during the study period with most states experiencing extremely high losses from floods than in other years. The periods of 1984–86 and 1996–99 also stand out from the yearly assessment and are categorized as high-flood-cost periods. This finding is consistent with previous studies reporting a larger increasing rate in flood hazard frequency than its magnitude in US^11–13, 30^. Although there are no evident trends of annual total precipitation and runoff, the extreme events have become more frequent towards the end of the study period (1983–99). Notably, the increasing trend in the frequency of 1-day extreme events coincides with the high flood damage events. Meanwhile, GDP exhibits an upward trend in most states over time while population remains relatively stable, with largest increase in the state of California, Texas and New York by 149%, 128% and 14% respectively. Concurrently, the areas of urban and crop lands have increased considerably in several states since 1955, with California, Texas and Florida ranked as the top three experiencing largest urbanland growth, while Texas, Kansas and North Dakota ranked as the top three states in cropland.

### Empirical relations between hazard and flood damage

**Figure 4:**
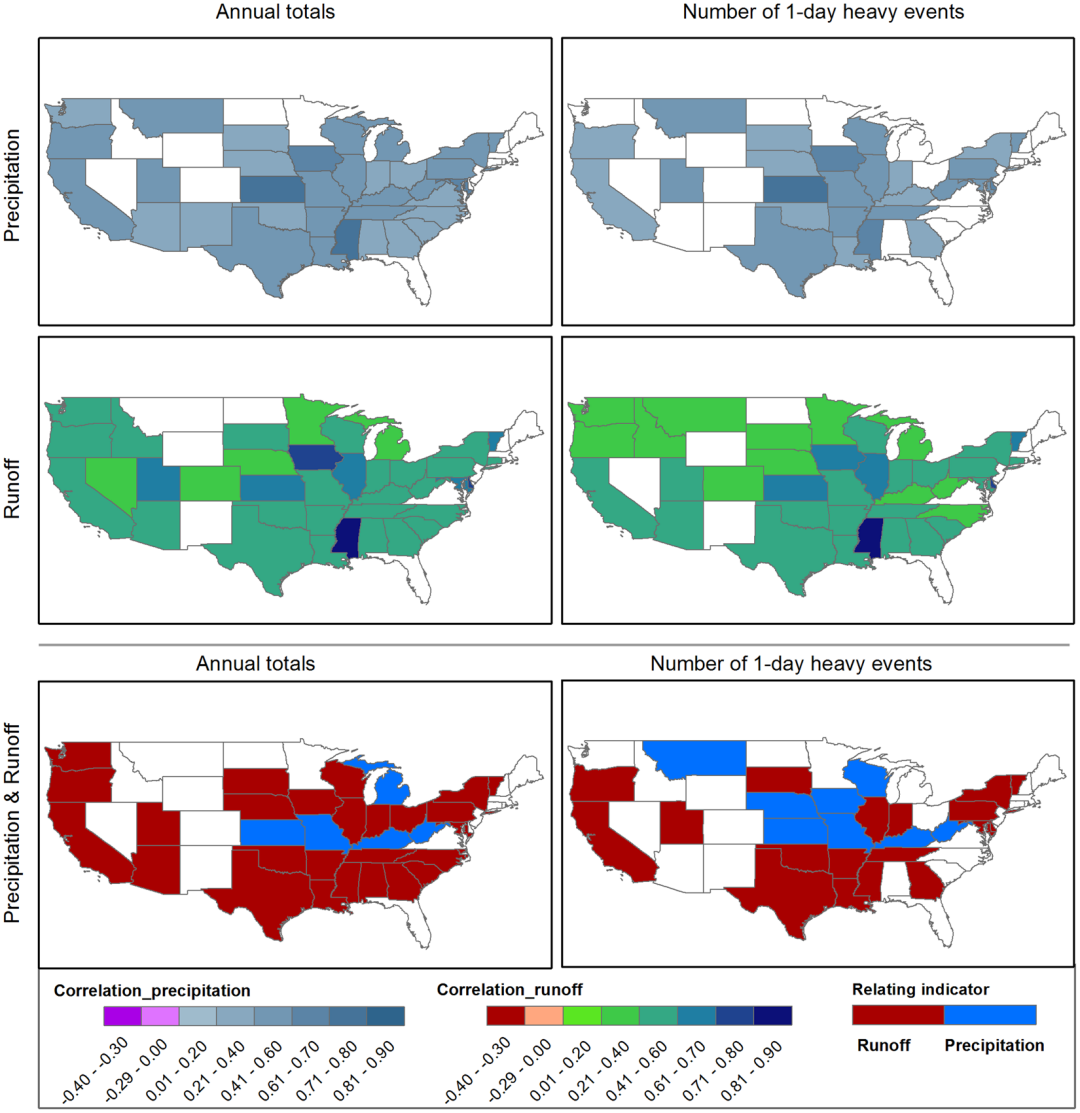
Correlation coefficient between hazard (indicated by annual total and extreme precipitation and runoff) and log-transformed flood damage. Only states where statistically significant relations are found at the 90% confidence level are shown. Figure was created using software ArcGIS 10.1 (http://www.esri.com/software/arcgis).

Overall, flood damage has exerted an increasing trend during the study period, and the change pattern of which resembles well those of extreme weather events, exposure and vulnerability variables as shown in Fig. 3. Next, we show the quantitative relations between flood damage and the potential influencing factors. For most states, there are significant correlations between hazards as indicated by precipitation and runoff extremes and flood damage (Fig. 4). This indicates that variations of flood damage can be well explained by hazards, which is in line with the findings from *Pielke and Downton* ^14^ and *Karl and Knight* ^31^. Nevertheless, in North Dakota, Wyoming, New Mexico, Nevada, Florida and Maine, the relations between flood damage and hazard are weak. This implies that variation of flood damage in those states could be mainly attributed to social-economic conditions, such as growth in wealth and demographic shifts.

Notably, the results show a strong correlation between flood damage and runoff. Specifically, there are 32 states (highlighted in Fig. 4) out of 48 where flood damage can be explained significantly by both precipitation and runoff totals, in which up to 84% of damage variation can be better explained by annual total runoff. As for the 1-day extreme events, statistically significant relations are found in 26 states, 73% of which (19 states) have stronger correlations between flood damage and runoff than precipitation. In extreme cases, the correlation coefficient between extreme runoff events and flood damage is up to 0.9 in the state of Mississippi, followed by Kansas, Iowa and Illinois. That is, runoff-indexed hazards have comparable power in explaining flood damage and can even outperform precipitation in certain regions. This has great implications for better understanding the variations in flood damage as runoff related indicators have been neglected in previous studies.

**Figure 5:**
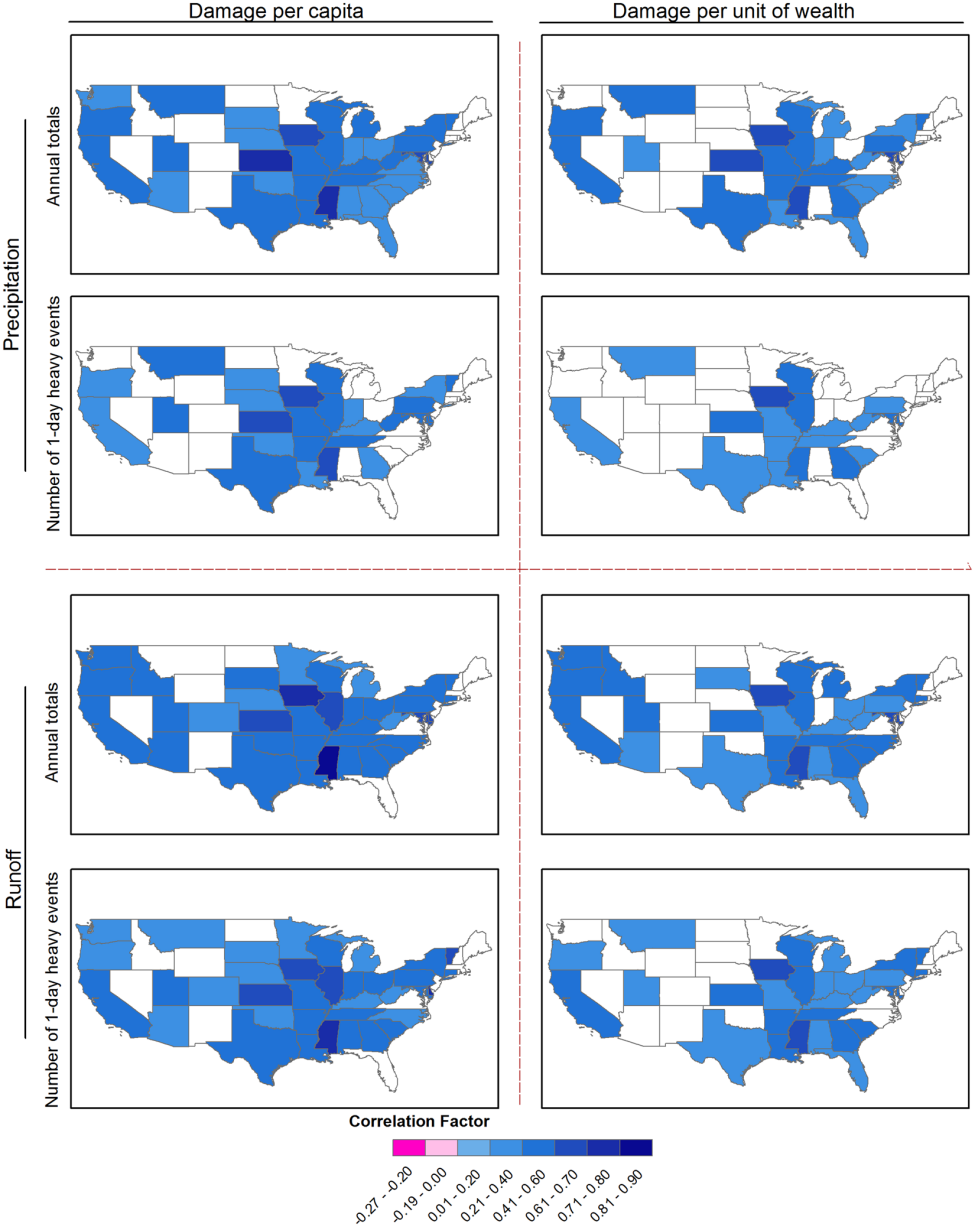
Correlation coefficient between hazard (indicated by annual total and extreme precipitation and runoff) and log-transformed Damage per capita (DPC) and Damage per unit of wealth (DPW). Only states where statistically significant relations are found at the 90% confidence level are shown. Figure was created using software ArcGIS 10.1 (http://www.esri.com/software/arcgis).

Similar spatial patterns are found for the relations between hazard indicators and the other two damage categories (i.e., DPC and DPW) in Fig. 5. Specifically, population variations are shown to have little impacts on the relations between flood hazard and damage^14^ as indicated by the similar spatial correlation patterns based on DPC (which excluded the population effects) and that based on total damage (i.e., D without excluding the population effects). However, this should not demise the important contribution of population to flood damage as demonstrated in previous studies^9, 35^. But rather, this indicates that the interannual variability of population has minor effects on the correlations between flood damage and hazard indicators. Based on DPW, states with statistically significant relations between flood damage and hazard indicators decreased considerably, demonstrating the role of social wealth growth (i.e., GDP) in regulating flood damage to physical hazards. In general, DPC and DPW are more correlated with runoff indicators for most states, except for Mississippi, Kansas, Iowa, Texas and Montana based on DPW, confirming the importance of considering runoff extremes as hazard indicators.

### The role of exposure and vulnerability in regulating flood damage

**Figure 6:**
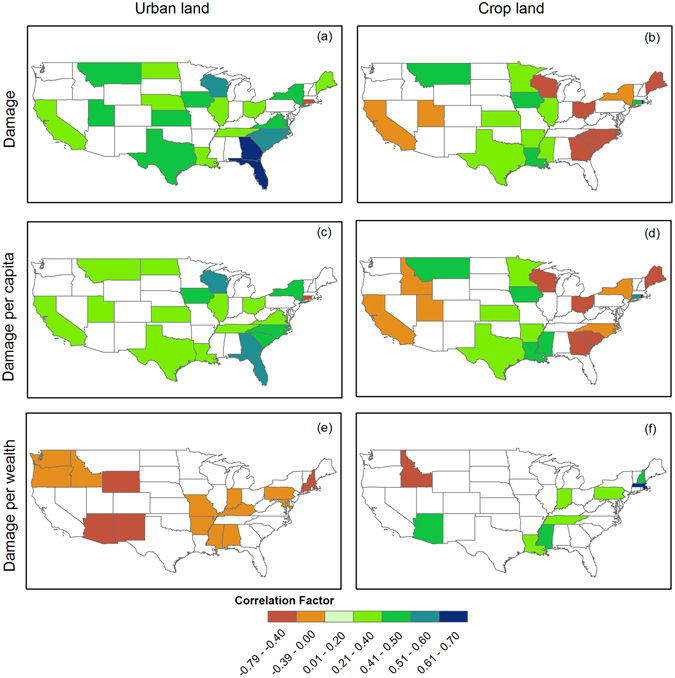
Correlation coefficient between urbanland and cropland areas and log-transformed damage (D), damage per capita (DPC) and damage per unit of wealth (DPW). Only states where statistically significant relations are found at the 90% confidence level are shown. Figure was created using software ArcGIS 10.1 (http://www.esri.com/software/arcgis).

Figure 6 shows the correlation coefficients of D, DPC and DPW with urbanland and cropland, which have not been explored in previous studies. The positive relations indicate that regional exposure and vulnerability contribute to the increasing damage and vice versa for negative relations. Indeed, urbanized areas are often associated with a stronger economy and more valuable assets, which increase the exposure to flood hazards and result in potentially higher flood damage^36, 37^. It is found that D is positively related to urban land in investigated states except for Connecticut and Rhode Island, especially in Florida and Georgia, indicating that flood damage in most states has exhibited an increasing trend along with urbanization. This is consistent with findings from *Kunkel et al*.^38^, *Barnolas and Llasat* ^39^, and *Huong and Pathirana* ^40^ that emphasized the contribution of urban development to the increasing flood damage in various regions worldwide. As for cropland, a noticeable regional difference is found with positive correlations in central regions and negative relationships in the rest areas. A possible explanation is that central states are primarily agriculture dominant regions where extreme floods can have direct impacts on crop growth, leading to agriculture production losses. Accounting for population effects has little impacts on the revealed patterns (Fig. 6c and d).

Interestingly, the correlation pattern changed considerably after accounting for the effects of GDP (Fig. 6e and f). For example, the states originally with positive relations in Fig. 6a became statistically insignificant after excluding the effects of wealth variations. Meanwhile, 16 other states where no significant correlations were found show negative relations with urbanland based on DPW. That is, given an identical hazard, flood damage is a combined result of both exposure (wealth/costs) and vulnerability (defense capability) (Fig. 1). Here, it is shown that growth of social wealth (i.e., GDP) played an important role in contributing to the increasing flood damage in US. After removing the effects of wealth growth, the role of urbanization in mitigating flood impacts is revealed. The changes in relations are in line with previous findings on ‘safe development paradox’ discussed in e.g., *Burby* ^37^, *Kates et al*.^36^ and *Cutter and Emrich* ^41^. Indeed, on the one hand, urban areas have more concentrated population and assets exposed to flood hazards, thus exhibiting positive relations with total flood damage. On the other hand, urbanization can enhance regional capability to cope with floods through upgrading of flood protection facilities and drainage systems in cities, thus implying a negative relation with flood damage. In fact, after excluding the effects of social wealth exposed to floods, the mitigation role of urbanland and related defensive capacity are revealed. This least understood aspect of hazard-exposure-vulnerability in urban areas has important implications for understanding the mechanisms behind flood damage.

### Predictability of flood damage at the state level

**Figure 7:**
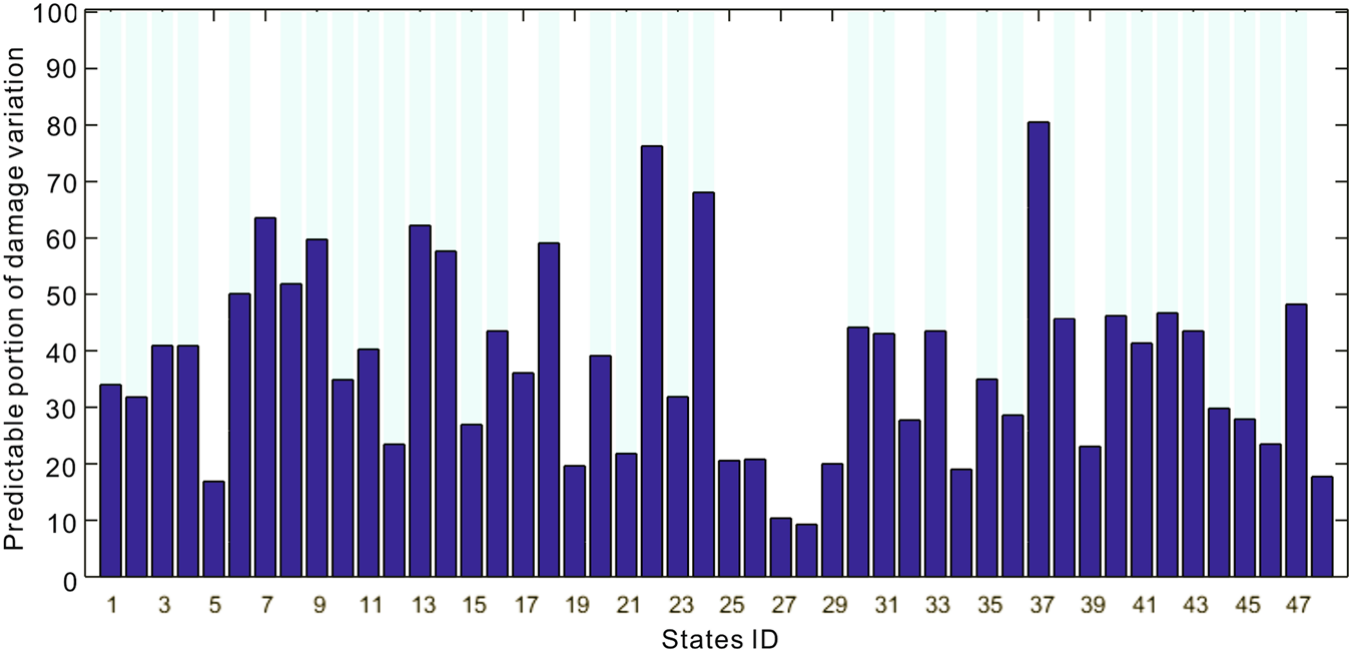
Portion of flood damage variation explained by the statistical model. The light blue bar indicates the state where flood damage variations can be predicted significantly at the 90% confidence level. Figure was created using software MATLAB 2015a (http://www.mathworks.com/).

Based upon the above analyses, we then construct multivariate regression models for each state, with flood damage as the dependent variable, and hazard, exposure and vulnerability indicators as independent variables. Here, the most highly related indicator (precipitation versus runoff, annual totals versus the 1-day extreme events) is selected as the hazard variable, while socio-economic indicators (i.e., GDP, POP, urban and crop lands) are used. Figure 7 shows the performance of established statistical models in explaining the variations of flood damage based on those indicators for each state. The light blue bar indicates that the relation is statistically significant at the 90% confidence level. In total, flood damage can be predicted in 34 of 48 investigated states at the 90% confidence level. The inability to build significant relationships in the remaining 14 states indicates that the methodology is not applicable in all regions. Therefore, more regionalized studies might improve the specifications of flood damage relation with hazard-exposure-vulnerability. Overall, 42% of flood damage variations can be explained by our statistical model for the country as a whole. In particular, 76% and 68% of flood damage variations can be predicted in the state of Mississippi and Montana, respectively, demonstrating the feasibility of predicting future flood damage under various climate change and social-economic scenarios.

## Discussion and Conclusion

Flood damage is influenced not only by climate variability, but also by non-climatic factors that shape regional exposure and vulnerability to weather extremes at various spatial scales. Understanding historical trends of flood damage and the underlying driving forces is important for better planning and adaptations. This study builds upon previous studies by including various hazard, exposure and vulnerability indicators for explaining US flood damage at a finer spatial scale, i.e., state level. Besides commonly used physical and socioeconomic factors, several important explanatory variables for explaining flood damage as ignored in previous studies are investigated. Statistical models are then established for each state, aiming to explore the predictability of flood damage.

It is found that runoff indicators have comparable power in explaining flood damage variations and can even outperform the commonly used precipitation ones in most of US states. In extreme cases, the correlation coefficient between runoff extremes and flood damage in the state of Mississippi is up to 0.9, followed by Kansas, lowa and Illinois. The wealth growth is more vital and complex in regulating flood damage than population. The cropland can be used as an important indicator for damage growth in central United States. Importantly, results suggest that urbanization can to certain degree mitigate regional flood hazards in certain states of US and thereby can be considered as a proxy measure of flood defense capability based on DPW. The results emphasize the importance of considering the least-understood role of runoff indexed hazards and regional defensive capability for a better understanding of flood damage variations.

Overall, flood damage in 34 of 48 investigated states can be explained significantly by the statistical model, but with large variations at the state level. In particular, 76% and 68% of flood damage can be predicted in the state of Mississippi and Montana, respectively, demonstrating the model feasibility to predict future flood damage. However, several assumptions have to be made for future predictions. For example, the non-stationary assumption on historical response function adopted in this study may not hold in the future as future socio-economic activities can not only effect the exposure and vulnerability^42, 43^ conditions, but also change the hydrological regime^44, 45^. Especially, impacts of future social dynamics on hydrological systems are highly unpredictable^42^. Examining the uncertainty arising from the non-stationarity assumption would be non-trivial, meriting a separate study. There are several approaches to explore this, e.g., by dividing the study period into two sub-periods, simulating flood damage based on the response function derived from the first sub-period with inputs from the second sub-period and comparing the simulations with observed records in the second period to examine the validity of the assumption. In addition, probabilistic methods could be used to deal with the variations in the response functions for future implications^46^. Also, socio-hydrological modeling approaches^42–44^ can be adopted in order to capture the complex interactions between social and hydrological processes. Nevertheless, the focus of this study is to explore the relation of flood damage to hazard, exposure and vulnerability in the study period 1955–1999, which plays a critical and fundamental role in future predictions.

The limitation on the accuracy and consistency of flood damage records should be acknowledged as the collection and processing of the data may suffer potential uncertainties. Specifically, individual damage estimate by small and moderate flood event is occasionally omitted or largely underestimated, which may introduce uncertainties and/or biased estimates in the total damage in reality. For example, the impacts of coastal flooding which can cause considerable damage to coastal communities along the coasts of US^47–49^ are not included in current flood damage records. *Moftakhari et al*.^50^ reported that the low-cost frequent floods could aggregate over time and the cumulative costs of such floods can even exceed the costs induced by the extreme but infrequent floods in coastal areas. Hence, more robust conclusions require longer time series and improved data quality to verify the trends and the statistical relationships obtained in the study and to investigate the contribution of coastal flooding to total flood damage. In addition, uncertainty in simulated runoff by the VIC model arising from e.g., model structure, parameter and forcing may propagate and affect the state-specific relations as constructed in this study.

Notwithstanding these limitations, the framework and methodology laid out in this study provide valuable tools for flood characterization based on historical records. The large spatial heterogeneity of flood damage as well as the distinct pattern of the underlying causes as revealed in this study demonstrates the importance to assess flood damage at a finer scale. Also it is important to note that, besides the commonly adopted indicators (i.e., GDP and POP), other types of land use or economic indicators can be used to explain the variability in flood damage. However, to explore the causes of increasing flood damage is extremely difficult due to lack of data on population and economy development within floodplains, and the information on the performance of various flood control and management measures across US. Based on the developed geospatial datasets on natural hazards, population, wealth, cropland and urban area, we highlight the predictability of US state level flood damage and emphasize the importance of accounting for the least-understood effects of runoff, urbanland and cropland in conditioning flood damage, which have been neglected in previous studies.

## Materials and Methods

### Conceptual framework

Figure 1 shows the conceptual framework adopted in this study for exploring the underlying causes of flood damage. Specifically, precipitation extremes associated with climate variability/change could exert major impacts on flood damage^18, 51^. Yet, in the long term, changes in geographical and land surface characteristics of an area due to frequent flooding can lead to transformation of city land use and assets relocation to avoid disruptions of service and damage^2^. Urbanization and economic growth are considered to be the most common causes of increased exposure and can affect the level of vulnerability^22, 40, 52–54^. Meanwhile, humans and related socio-economic developments are likely to affect the hydrological regime^42^, surface routing process and planning of countermeasures, and thus have a secondary effect on hazard^55^. Effective flood control policies can enhance flood resilience system such that hazard (e.g., runoff), exposure and vulnerability could be reduced.

Generally, flood damage can be described as a joint function of hazard, exposure and vulnerability^22, 49^. Here, hazard characterizes the natural occurrence and magnitude of a damage-producing flood resulting from weather extremes, without human interference. In addition to the commonly adopted precipitation-related indicators (e.g., number of wet days, precipitation totals), we consider the use of runoff indicators (e.g., 1_day runoff, annual totals) as proxy of the combined effects of climatic and land surface conditions. Exposure is the expected number of population and economic assets exposed to the hazardous conditions in flood-prone areas. In this study, exposure mainly characterizes the population, landuse and socioeconomic conditions. Specially, we include areas of urban and crop lands as measures of exposure besides the two widely adopted indicators (GDP and POP). Vulnerability is the susceptibility of exposed elements to flood hazard. Here, vulnerability is mainly considered to include man-made efforts to mitigate the impacts of natural flood hazards on exposure^10, 22^, e.g., defense capability (i.e., the ability to mitigate the hazard impacts) (Fig. 1). That is, places with both low exposure and low vulnerability (i.e., high defense capacities) are less adversely affected by flood hazard. To address the gap on the relations between flood damage and regional defense capacity, we use the urbanland and its interactions with exposure indicators to reflect the vulnerability level of the system under threat, due to the absence of other relevant observations.

### Data

State level flood damage records for the period 1955–99 are obtained from the National Weather Service (NWS), which is responsible for maintaining long-term flood damage statistics. The NWS damage records refer to the direct economic damage (including loss of property and crops, costs of repairing damaged building and roads) caused by significant flood events due to rainfall and/or snowmelt^14^. The damage data are compiled right after each major flood event and updated accordingly in the dataset. Annual flood damage is provided for each hydrological year from 1 October to 30 September of the following year in thousands of current dollars. Though the NWS estimates are obtained from various sources, data quality is argued to be sufficient for use in trend analysis^14^.

Historical daily climate including precipitation is obtained from *Maurer et al*.^56^ which provides gridded products at 0.125 degree across the conterminous United States. Daily runoff is simulated using the Variable Infiltration Capacity (VIC) model^57, 58^ driven by observed climate. The VIC model is a macro scale hydrologic model accounting for subgrid-scale variability and has been widely used at the regional and global scale^59–62^. The annual total and maximum 1-day extreme precipitation and runoff are calculated at each grid cell and then aggregated into the state level for our analysis.

Four socio-economic factors are selected. The state-level POP and GDP data are obtained from the US’s Department of Commerce, Census Bureau (https://www.census.gov/) and Bureau of Economic analysis (BEA, http://www.bea.gov/), respectively. The population is provided for 1 July of each year, in thousands. The GDP estimates are given for 31 December of each year, in millions of current dollars. The USDA (United States Department of Agriculture, http://www.usda.gov/) provides major land use data for the past 50 years, including forestland, grassland pasture and rangeland, cropland, special uses (primarily parks and wildlife areas), miscellaneous uses (like tundra or swamps) and urban land. Here, areas of urban and crop lands for the same period, in thousands of acres, are obtained for use.

### Analysis

The damage estimates are first standardized following *Pielke and Downton* ^14^, *Barredo* ^35^, and *Changnon and Changnon* ^63^. The inflation-adjusted damage data are then normalized by GDP and POP to minimize the time-variant socio-economic influences on damage estimates. Here, three categories of flood damage are used in the subsequent analysis: total annual damage (D), damage per capita (DPC), and damage per unit of GDP (DPW), such that the relative impacts of other indicators on the damage can be quantified while controlling the population and wealth effects. For example, the total annual damage is likely to be higher in states with high level of wealth, such as California given the same level of hazard.

Statistic distributions of selected indicators are examined by calculating the range, mean, median and standard deviation (STDEV). Due to the large variation in the damage estimates, log transformation is used for all the three damage categories (i.e., D, DPC and DPW) for better fitting the normal distribution. Statistical analysis is then conducted by investigating the relations between log-transformed flood damage and selected indicators using the software package Matlab 2015a. Pearson correlation coefficients are computed for all pairwise variable combinations based on their linear dependence. The statistical significance of the linear trend is tested at the 90% confidence level. The correlation analysis is conducted to select appropriate hazard and vulnerability indicators for construction of statistical model for predicting flood damage. Multi-variable linear regression is subsequently employed to establish the flood damage model based on selected indicators.

## Electronic supplementary material

Supplementary materials
