# Supplementary material for: Predictability of state-level flood damage in the conterminous United States: the role of hazard, exposure and vulnerability
Source: Sci Rep. 2017 Jul 13;7:5354. doi: 10.1038/s41598-017-05773-4 (PMC5509717; doi:10.1038/s41598-017-05773-4)

Predictability of state-level flood damage in the conterminous United States: the role of hazard, exposure and vulnerability

Qianqian Zhou[1,2], Guoyong Leng[2,*] and Leyang Feng[2]

[1]School of Civil and Transportation Engineering, Guangdong University of Technology, Waihuan Xi Road, Guangzhou 510006, China

[2]Joint Global Change Research Institute, Pacific Northwest National Laboratory, College Park MD, USA

*Corresponding author address: Guoyong Leng, Joint Global Change Research Institute, Pacific Northwest National Laboratory, College Park MD, 20740.

E-mail: Guoyong.leng@pnnl.gov

Supplementary Materials

Table S1: State-level (a) mean annual flood damage (in thousands of current dollars), (b) the standard deviation of flood damage normalized by the mean (i.e., coefficient of variation, CV), (c) median GDP (in millions of current dollars) and (d) median GDP/median POP (in thousands) for the period of 1955-99.

Table S2: Correlation coefficient between hazard (indicated by annual total and extreme precipitation and runoff) and log-transformed flood damage (D).

Table S3: Correlation coefficient between hazard (indicated by annual total and extreme precipitation and runoff) and Damage per capita (DPC).

Table S4: Correlation coefficient between hazard (indicated by annual total and extreme precipitation and runoff) and Damage per unit of wealth (DPW).

Table S5: Correlation coefficient between urbanland and cropland areas and log-transformed damage (D).

Table S6: Correlation coefficient between urbanland and cropland areas and DPC.

Table S7: Correlation coefficient between urbanland and cropland areas and DPW.

Table S8: Portion of flood damage variation explained by the statistical model.

Figure S1: Temporal changes in flood damage, GDP and total annual runoffs during the study period.

Table S1: State-level (a) mean annual flood damage (in thousands of current dollars), (b) the standard deviation of flood damage normalized by the mean (i.e., coefficient of variation, CV), (c) median GDP (in millions of current dollars) and (d) median GDP/median POP (in thousands) for the period of 1955-99.

STATE_ NAME	STATE_ Abbreviation	State_ id	Damage_ Mean	damage_ CV(%)	GDP_ Median	GDP/POP_ Median
**Alabama**	AL	1	20336	313.1	26725	7.070
**Arizona**	AZ	2	25889	239.4	19609	8.086
**Arkansas**	AR	3	24438	333.9	15182	6.873
**California**	CA	4	178588	234.8	229592	10.272
**Colorado**	CO	5	38048	271.0	25428	9.432
**Connecticut**	CT	6	23574	345.0	29598	9.591
**Delaware**	DE	7	605	231.1	6056	10.230
**Florida**	FL	8	36867	225.5	65272	7.370
**Georgia**	GA	9	17839	316.8	40785	7.813
**Idaho**	ID	10	27438	409.7	7206	8.161
**Illinois**	IL	11	92640	438.9	115077	10.107
**Indiana**	IN	12	19953	128.1	47901	8.828
**Iowa**	IA	13	191031	474.8	27011	9.623
**Kansas**	KS	14	31935	289.1	20539	8.849
**Kentucky**	KY	15	30376	255.8	29176	8.163
**Louisiana**	LA	16	184471	309.4	39670	9.883
**Maine**	ME	17	7286	183.8	7647	6.927
**District of Columbia**	DC	18	16304	277.6	35555	7.337
**Maryland**	MD	18	16304	277.6	35555	7.337
**Massachusetts**	MA	19	22078	177.1	50719	8.808
**Michigan**	MI	20	22492	349.7	89326	9.800
**Minnesota**	MN	21	58175	329.2	36830	9.233
**Mississippi**	MS	22	39683	341.7	15957	6.489
**Missouri**	MO	23	108572	485.3	41150	8.462
**Montana**	MT	24	5359	221.6	6350	8.247
**Nebraska**	NE	25	21607	229.8	13874	8.911
**Nevada**	NV	26	23380	490.2	7416	10.938
**New Hampshire**	NH	27	3778	158.0	6298	7.239
**New Jersey**	NJ	28	72672	232.7	65839	8.970

**New Mexico**	NM	29	5012	167.5	10102	8.308
**New York**	NY	30	50034	259.5	179890	10.067
**North Carolina**	NC	31	91418	551.6	43822	7.707
**North Dakota**	ND	32	154147	417.2	5243	8.192
**Ohio**	OH	33	23119	146.5	96995	9.020
**Oklahoma**	OK	34	37314	356.7	24022	8.370
**Oregon**	OR	35	93188	536.0	22047	9.010
**Pennsylvania**	PA	36	111596	404.4	99024	8.359
**Rhode Island**	RI	37	3268	258.5	7245	7.610
**South Carolina**	SC	38	3604	209.1	20103	6.719
**South Dakota**	SD	39	41960	328.7	5116	7.404
**Tennessee**	TN	40	10009	158.6	33598	7.596
**Texas**	TX	41	146408	227.1	130404	9.884
**Utah**	UT	42	29222	382.9	10381	7.864
**Vermont**	VT	43	8044	210.1	3347	6.803
**Virginia**	VA	44	60290	247.7	43219	8.323
**Washington**	WA	45	24284	285.1	36826	9.753
**West Virginia**	WV	46	32515	317.4	14439	7.938
**Wisconsin**	WI	47	49280	324.3	40686	8.793
**Wyoming**	WY	48	2624	321.2	5540	13.414

Table S2: Correlation coefficient between hazard (indicated by annual total and extreme precipitation and runoff) and log-transformed flood damage (D).

STATE	ID	Precipitation				Runoff			
		Annual totals		Number of 1-day events		Annual totals		Number of 1-day events	
		C	***p***	***C***	***p***	***C***	***p***	***C***	***p***
**AL**	1	0.3214	0.0491	0.2391	0.1483	0.4891	0.0018	0.5536	0.0003
**AZ**	2	0.3719	0.0303	0.2037	0.2479	0.4802	0.0040	0.4047	0.0176
**AR**	3	0.5489	0.0002	0.4574	0.0030	0.5958	0.0001	0.5686	0.0001
**CA**	4	0.4776	0.0021	0.3835	0.0160	0.5251	0.0006	0.5697	0.0002
**CO**	5	0.2670	0.1465	0.2208	0.2326	0.3384	0.0626	0.3991	0.0261
**CT**	6	0.3736	0.1047	0.1009	0.6722	0.5378	0.0145	0.5758	0.0079
**DE**	7	0.5343	0.1384	0.6308	0.0685	0.7090	0.0325	0.7612	0.0172
**FL**	8	0.2742	0.1056	0.2734	0.1067	0.2602	0.1253	0.2232	0.1906
**GA**	9	0.3734	0.0249	0.3635	0.0293	0.5371	0.0007	0.5140	0.0013
**ID**	10	0.1362	0.4574	0.0750	0.6832	0.5111	0.0028	0.3162	0.0778
**IL**	11	0.5914	0.0000	0.5962	0.0000	0.6192	0.0000	0.6152	0.0000
**IN**	12	0.3785	0.0135	0.3333	0.0310	0.4345	0.0040	0.4607	0.0021
**IA**	13	0.6778	0.0000	0.6935	0.0000	0.7030	0.0000	0.6746	0.0000
**KS**	14	0.7321	0.0000	0.7018	0.0000	0.6569	0.0000	0.6467	0.0000
**KY**	15	0.4744	0.0020	0.3908	0.0127	0.4128	0.0081	0.3604	0.0223
**LA**	16	0.4131	0.0136	0.3153	0.0650	0.5192	0.0014	0.5090	0.0018
**ME**	17	-0.0522	0.8222	0.0533	0.8184	0.2058	0.3708	0.1385	0.5493
**DC**	18	0.6895	0.0001	0.5684	0.0020	0.6923	0.0001	0.5708	0.0019
**MD**	18	0.6895	0.0001	0.5684	0.0020	0.6923	0.0001	0.5708	0.0019
**MA**	19	-0.2626	0.3259	-0.5468	0.0284	0.0291	0.9148	0.0334	0.9022
**MI**	20	0.4078	0.0479	0.3415	0.1025	0.3796	0.0673	0.3518	0.0918
**MN**	21	0.1945	0.2420	0.2119	0.2015	0.3651	0.0242	0.3323	0.0415
**MS**	22	0.7580	0.0000	0.6639	0.0000	0.8357	0.0000	0.8009	0.0000
**MO**	23	0.5488	0.0002	0.5025	0.0008	0.5197	0.0005	0.4638	0.0023
**MT**	24	0.5162	0.0025	0.4091	0.0201	0.2017	0.2683	0.3584	0.0440
**NE**	25	0.3192	0.0541	0.3851	0.0186	0.3924	0.0163	0.3448	0.0366
**NV**	26	0.0347	0.8608	0.0046	0.9814	0.3635	0.0573	0.3036	0.1162
**NH**	27	0.1472	0.5600	0.1798	0.4753	0.2895	0.2440	0.1965	0.4345
**NJ**	28	0.0898	0.6837	-0.1209	0.5825	0.2175	0.3188	0.2119	0.3318
**NM**	29	0.3357	0.0936	0.2409	0.2359	0.2003	0.3266	-0.0219	0.9153

**NY**	30	0.4503	0.0066	0.3754	0.0263	0.5771	0.0003	0.5411	0.0008
**NC**	31	0.3237	0.0542	0.1318	0.4434	0.4571	0.0051	0.3230	0.0547
**ND**	32	0.1459	0.4589	0.0168	0.9323	0.2689	0.1665	0.1799	0.3597
**OH**	33	0.3014	0.0587	0.2303	0.1528	0.4648	0.0025	0.4050	0.0095
**OK**	34	0.3899	0.0171	0.3545	0.0313	0.4294	0.0080	0.4169	0.0103
**OR**	35	0.4671	0.0027	0.3008	0.0628	0.5689	0.0002	0.3890	0.0144
**PA**	36	0.5077	0.0011	0.4053	0.0116	0.5147	0.0009	0.5007	0.0014
**RI**	37	-0.3896	0.3401	-0.6273	0.0959	-0.0175	0.9671	-0.1588	0.7072
**SC**	38	0.2914	0.0847	0.2435	0.1525	0.4494	0.0060	0.4514	0.0057
**SD**	39	0.3897	0.0275	0.3517	0.0484	0.4421	0.0113	0.3962	0.0248
**TN**	40	0.5510	0.0003	0.4752	0.0022	0.5708	0.0001	0.5086	0.0009
**TX**	41	0.4615	0.0024	0.4249	0.0056	0.4926	0.0011	0.4778	0.0016
**UT**	42	0.4479	0.0070	0.4592	0.0055	0.6111	0.0001	0.4782	0.0037
**VT**	43	0.5408	0.0114	0.4356	0.0484	0.6094	0.0034	0.6503	0.0014
**VA**	44	0.3152	0.0740	0.2709	0.1273	0.2531	0.1553	0.2168	0.2256
**WA**	45	0.2958	0.0755	0.2155	0.2002	0.4575	0.0044	0.3691	0.0246
**WV**	46	0.4808	0.0015	0.4607	0.0024	0.4007	0.0094	0.3851	0.0129
**WI**	47	0.5083	0.0041	0.4838	0.0068	0.5247	0.0029	0.4438	0.0140
**WY**	48	0.2876	0.1730	0.2401	0.2583	-0.0381	0.8598	-0.0352	0.8704

C :correlation coefficients; ***p*** : p-values

Table S3: Correlation coefficient between hazard (indicated by annual total and extreme precipitation and runoff) and Damage per capita (DPC).

STATE	ID	Precipitation				Runoff			
		Annual totals		Number of 1-day events		Annual totals		Number of 1-day events	
		C	***p***	***C***	***p***	***C***	***p***	***C***	***p***
**AL**	1	0.3123	0.0563	0.2240	0.1765	0.4781	0.0024	0.5462	0.0004
**AZ**	2	0.3490	0.0431	0.1681	0.3419	0.4469	0.0081	0.3552	0.0392
**AR**	3	0.5482	0.0003	0.4486	0.0037	0.5895	0.0001	0.5598	0.0002
**CA**	4	0.4837	0.0018	0.3716	0.0198	0.5374	0.0004	0.5634	0.0002
**CO**	5	0.2318	0.2096	0.1709	0.3581	0.3107	0.0889	0.3551	0.0500
**CT**	6	0.3720	0.1063	0.0901	0.7055	0.5394	0.0141	0.5726	0.0083
**DE**	7	0.5036	0.1670	0.5968	0.0898	0.6818	0.0431	0.7330	0.0246
**FL**	8	0.2868	0.0899	0.2710	0.1099	0.2731	0.1071	0.2439	0.1517
**GA**	9	0.3938	0.0175	0.3808	0.0220	0.5517	0.0005	0.5222	0.0011
**ID**	10	0.1303	0.4773	0.0552	0.7640	0.5035	0.0033	0.2833	0.1162
**IL**	11	0.5911	0.0000	0.5934	0.0000	0.6157	0.0000	0.6119	0.0000
**IN**	12	0.3720	0.0153	0.3242	0.0362	0.4234	0.0052	0.4499	0.0028
**IA**	13	0.6777	0.0000	0.6931	0.0000	0.7024	0.0000	0.6742	0.0000
**KS**	14	0.7303	0.0000	0.6972	0.0000	0.6520	0.0000	0.6434	0.0000
**KY**	15	0.4713	0.0021	0.3851	0.0141	0.4084	0.0089	0.3578	0.0234
**LA**	16	0.4145	0.0133	0.3153	0.0651	0.5174	0.0015	0.5061	0.0019
**ME**	17	-0.0420	0.8565	0.0584	0.8013	0.2203	0.3372	0.1539	0.5053
**DC**	18	0.6878	0.0001	0.5604	0.0024	0.6882	0.0001	0.5656	0.0021
**MD**	18	0.6878	0.0001	0.5604	0.0024	0.6882	0.0001	0.5656	0.0021
**MA**	19	-0.2583	0.3341	-0.5478	0.0281	0.0335	0.9019	0.0349	0.8979
**MI**	20	0.4061	0.0489	0.3357	0.1088	0.3825	0.0651	0.3534	0.0902
**MN**	21	0.1875	0.2596	0.2027	0.2223	0.3571	0.0278	0.3224	0.0484
**MS**	22	0.7552	0.0000	0.6567	0.0000	0.8307	0.0000	0.7952	0.0000
**MO**	23	0.5420	0.0003	0.4933	0.0010	0.5087	0.0007	0.4522	0.0030
**MT**	24	0.5200	0.0023	0.4032	0.0221	0.2078	0.2538	0.3563	0.0453
**NE**	25	0.3135	0.0588	0.3796	0.0205	0.3851	0.0186	0.3361	0.0420
**NV**	26	-0.0050	0.9798	-0.0488	0.8051	0.3069	0.1122	0.2213	0.2577
**NH**	27	0.1431	0.5710	0.1726	0.4934	0.2766	0.2665	0.1885	0.4538
**NJ**	28	0.0889	0.6867	-0.1284	0.5593	0.2202	0.3127	0.2137	0.3276
**NM**	29	0.2763	0.1718	0.1742	0.3946	0.1332	0.5166	-0.0834	0.6853

**NY**	30	0.4486	0.0069	0.3737	0.0270	0.5773	0.0003	0.5418	0.0008
**NC**	31	0.3315	0.0483	0.1310	0.4464	0.4581	0.0050	0.3110	0.0649
**ND**	32	0.1460	0.4585	0.0165	0.9336	0.2688	0.1666	0.1797	0.3602
**OH**	33	0.3001	0.0599	0.2298	0.1537	0.4628	0.0026	0.4034	0.0098
**OK**	34	0.3780	0.0211	0.3379	0.0408	0.4136	0.0110	0.3978	0.0148
**OR**	35	0.4613	0.0031	0.2826	0.0813	0.5621	0.0002	0.3721	0.0197
**PA**	36	0.5055	0.0012	0.4036	0.0120	0.5123	0.0010	0.4989	0.0014
**RI**	37	-0.3776	0.3565	-0.6222	0.0995	-0.0065	0.9877	-0.1491	0.7246
**SC**	38	0.3102	0.0656	0.2613	0.1236	0.4646	0.0043	0.4604	0.0047
**SD**	39	0.3858	0.0292	0.3478	0.0511	0.4384	0.0121	0.3924	0.0263
**TN**	40	0.5528	0.0003	0.4738	0.0023	0.5737	0.0001	0.5124	0.0009
**TX**	41	0.4652	0.0022	0.4167	0.0067	0.4851	0.0013	0.4699	0.0019
**UT**	42	0.4214	0.0117	0.4268	0.0106	0.5808	0.0003	0.4440	0.0075
**VT**	43	0.5312	0.0132	0.4237	0.0556	0.5956	0.0044	0.6416	0.0017
**VA**	44	0.3100	0.0791	0.2599	0.1440	0.2440	0.1711	0.2044	0.2538
**WA**	45	0.2875	0.0844	0.1981	0.2398	0.4591	0.0043	0.3514	0.0330
**WV**	46	0.4800	0.0015	0.4598	0.0025	0.3994	0.0097	0.3847	0.0130
**WI**	47	0.5103	0.0040	0.4828	0.0069	0.5275	0.0027	0.4476	0.0131
**WY**	48	0.2814	0.1829	0.2233	0.2943	-0.0558	0.7958	-0.0626	0.7712

C :correlation coefficients; ***p*** : p-values

Table S4: Correlation coefficient between hazard (indicated by annual total and extreme precipitation and runoff) and Damage per unit of wealth (DPW).

STATE	ID	Precipitation				Runoff			
		Annual totals		Number of 1-day events		Annual totals		Number of 1-day events	
		C	*p*	*C*	*p*	*C*	*p*	*C*	*p*
**AL**	1	0.1729	0.2991	0.0371	0.8251	0.2934	0.0738	0.3864	0.0166
**AZ**	2	0.2689	0.1240	0.0790	0.6569	0.3391	0.0498	0.2217	0.2076
**AR**	3	0.4520	0.0034	0.2978	0.0620	0.4447	0.0040	0.4015	0.0102
**CA**	4	0.4711	0.0025	0.2963	0.0670	0.5467	0.0003	0.4986	0.0012
**CO**	5	0.0894	0.6326	-0.0200	0.9149	0.1894	0.3075	0.1697	0.3613
**CT**	6	0.3484	0.1322	0.0240	0.9198	0.4959	0.0262	0.5132	0.0207
**DE**	7	0.3317	0.3831	0.3840	0.3076	0.5347	0.1380	0.5748	0.1055
**FL**	8	0.3045	0.0710	0.2422	0.1546	0.2899	0.0863	0.2855	0.0914
**GA**	9	0.4634	0.0044	0.4325	0.0084	0.5625	0.0004	0.4985	0.0020
**ID**	10	0.1014	0.5808	-0.0241	0.8957	0.4321	0.0135	0.1343	0.4637
**IL**	11	0.5462	0.0002	0.4954	0.0009	0.5043	0.0007	0.5103	0.0006
**IN**	12	0.2596	0.0969	0.1853	0.2400	0.2394	0.1268	0.2683	0.0859
**IA**	13	0.6707	0.0000	0.6554	0.0000	0.6765	0.0000	0.6503	0.0000
**KS**	14	0.6223	0.0000	0.5586	0.0002	0.4970	0.0013	0.5182	0.0007
**KY**	15	0.4115	0.0083	0.3023	0.0580	0.3374	0.0333	0.3044	0.0562
**LA**	16	0.3936	0.0193	0.2847	0.0974	0.4643	0.0050	0.4437	0.0076
**ME**	17	0.0632	0.7853	0.1016	0.6614	0.3418	0.1294	0.2925	0.1982
**DC**	18	0.6256	0.0005	0.4528	0.0177	0.6075	0.0008	0.4837	0.0106
**MD**	18	0.6256	0.0005	0.4528	0.0177	0.6075	0.0008	0.4837	0.0106
**MA**	19	-0.1905	0.4797	-0.5309	0.0343	0.0793	0.7704	0.0496	0.8554
**MI**	20	0.3995	0.0531	0.2751	0.1932	0.4276	0.0372	0.3881	0.0609
**MN**	21	0.1153	0.4907	0.1091	0.5142	0.2674	0.1046	0.2197	0.1851
**MS**	22	0.6187	0.0000	0.4827	0.0016	0.6681	0.0000	0.6396	0.0000
**MO**	23	0.4166	0.0067	0.3373	0.0310	0.3365	0.0315	0.2799	0.0763
**MT**	24	0.5200	0.0023	0.3016	0.0935	0.2838	0.1155	0.3081	0.0863
**NE**	25	0.1835	0.2769	0.2271	0.1765	0.2185	0.1940	0.1428	0.3993
**NV**	26	-0.0561	0.7766	-0.1179	0.5501	0.2072	0.2901	0.0917	0.6427
**NH**	27	0.1146	0.6507	0.1275	0.6142	0.1916	0.4463	0.1372	0.5871
**NJ**	28	0.1110	0.6142	-0.1705	0.4367	0.2496	0.2507	0.2351	0.2802
**NM**	29	0.0340	0.8691	-0.0788	0.7020	-0.1263	0.5388	-0.2918	0.1480

**NY**	30	0.3773	0.0254	0.2667	0.1214	0.5242	0.0012	0.5004	0.0022
**NC**	31	0.3395	0.0428	0.1064	0.5370	0.4015	0.0152	0.1851	0.2798
**ND**	32	0.0746	0.7060	-0.0796	0.6871	0.2230	0.2541	0.1041	0.5979
**OH**	33	0.2534	0.1146	0.1785	0.2703	0.3654	0.0204	0.3058	0.0550
**OK**	34	0.2560	0.1262	0.1871	0.2675	0.2598	0.1205	0.2278	0.1751
**OR**	35	0.4062	0.0103	0.1891	0.2490	0.4983	0.0012	0.2790	0.0854
**PA**	36	0.4015	0.0125	0.3090	0.0591	0.3944	0.0143	0.3992	0.0130
**RI**	37	-0.2700	0.5179	-0.5832	0.1291	0.0669	0.8749	-0.0746	0.8606
**SC**	38	0.3872	0.0196	0.3462	0.0386	0.4878	0.0025	0.4428	0.0068
**SD**	39	0.2845	0.1145	0.2204	0.2254	0.3460	0.0524	0.2794	0.1215
**TN**	40	0.4746	0.0023	0.3847	0.0156	0.4994	0.0012	0.4579	0.0034
**TX**	41	0.4035	0.0089	0.3121	0.0470	0.3713	0.0169	0.3541	0.0231
**UT**	42	0.2852	0.0968	0.2710	0.1154	0.4176	0.0126	0.2838	0.0984
**VT**	43	0.4561	0.0377	0.3362	0.1362	0.4858	0.0256	0.5657	0.0075
**VA**	44	0.2577	0.1477	0.1750	0.3301	0.1707	0.3423	0.1121	0.5345
**WA**	45	0.2440	0.1455	0.1321	0.4359	0.4308	0.0078	0.2629	0.1160
**WV**	46	0.3861	0.0126	0.3696	0.0174	0.3056	0.0520	0.2940	0.0621
**WI**	47	0.4936	0.0056	0.4304	0.0176	0.5306	0.0026	0.4733	0.0083
**WY**	48	0.1924	0.3678	0.1019	0.6358	-0.1475	0.4914	-0.2228	0.2953

C :correlation coefficients; ***p*** : p-values

Table S5: Correlation coefficient between urbanland and cropland areas and log-transformed damage (D).

STATE	ID	Urban land		Crop land	
		C	*p*	*C*	*p*
**AL**	1	0.2682	0.1035	-0.2546	0.1230
**AZ**	2	0.0410	0.8180	0.0477	0.7889
**AR**	3	0.2465	0.1252	0.3801	0.0155
**CA**	4	0.3904	0.0140	-0.3828	0.0162
**CO**	5	0.1914	0.3024	0.0422	0.8217
**CT**	6	-0.4245	0.0621	0.4973	0.0257
**DE**	7	0.2226	0.5648	0.0062	0.9874
**FL**	8	0.6545	0.0000	0.2433	0.1527
**GA**	9	0.6063	0.0001	-0.4792	0.0031
**ID**	10	0.0640	0.7278	-0.2621	0.1473
**IL**	11	0.3281	0.0339	0.2676	0.0866
**IN**	12	0.2358	0.1328	-0.0792	0.6183
**IA**	13	0.4894	0.0018	0.4543	0.0042
**KS**	14	0.4020	0.0112	0.3955	0.0127
**KY**	15	0.0240	0.8833	0.0624	0.7023
**LA**	16	0.3228	0.0586	0.4757	0.0039
**ME**	17	0.3802	0.0891	-0.5517	0.0095
**DC**	18	0.0179	0.9293	-0.0975	0.6285
**MD**	18	0.0179	0.9293	-0.0975	0.6285
**MA**	19	-0.2549	0.3407	0.3627	0.1673
**MI**	20	0.2723	0.1979	-0.0264	0.9026
**MN**	21	0.2395	0.1475	0.3709	0.0219
**MS**	22	0.1553	0.3387	0.3953	0.0116
**MO**	23	0.1363	0.3956	0.1503	0.3482
**MT**	24	0.4188	0.0170	0.4896	0.0045
**NE**	25	0.2950	0.0763	0.1683	0.3194
**NV**	26	0.2343	0.2301	0.1125	0.5688
**NH**	27	0.0631	0.8037	-0.1291	0.6097
**NJ**	28	0.0310	0.8882	-0.1177	0.5927
**NM**	29	-0.0381	0.8533	-0.0612	0.7663
**NY**	30	0.4208	0.0118	-0.3925	0.0197

**NC**	31	0.5108	0.0015	-0.4392	0.0074
**ND**	32	0.3403	0.0764	0.2614	0.1791
**OH**	33	0.3524	0.0257	-0.4182	0.0072
**OK**	34	0.2203	0.1900	0.2680	0.1087
**OR**	35	0.0828	0.6163	-0.1595	0.3322
**PA**	36	-0.0190	0.9099	0.0011	0.9950
**RI**	37	-0.7922	0.0191	0.6883	0.0591
**SC**	38	0.5208	0.0011	-0.4918	0.0023
**SD**	39	0.2420	0.1820	0.1077	0.5573
**TN**	40	0.3719	0.0197	-0.2178	0.1828
**TX**	41	0.4853	0.0013	0.3495	0.0251
**UT**	42	0.4176	0.0126	-0.3252	0.0566
**VT**	43	0.1988	0.3877	-0.2536	0.2674
**VA**	44	0.4093	0.0180	0.1662	0.3553
**WA**	45	0.1780	0.2920	0.2692	0.1071
**WV**	46	0.0720	0.6546	0.0399	0.8045
**WI**	47	0.5268	0.0028	-0.4810	0.0071
**WY**	48	0.0173	0.9362	0.1588	0.4586

C :correlation coefficients; ***p*** : p-values

Table S6: Correlation coefficient between urbanland and cropland areas and DPC.

STATE	ID	Urban land		Crop land	
		C	***p***	***C***	***p***
**AL**	1	0.2184	0.1878	-0.2081	0.2100
**AZ**	2	-0.1237	0.4859	0.1859	0.2926
**AR**	3	0.1885	0.2441	0.3355	0.0343
**CA**	4	0.3087	0.0559	-0.3123	0.0529
**CO**	5	0.0916	0.6242	0.0230	0.9022
**CT**	6	-0.4465	0.0484	0.5224	0.0181
**DE**	7	0.1558	0.6889	0.0416	0.9154
**FL**	8	0.5518	0.0005	0.1845	0.2813
**GA**	9	0.5433	0.0006	-0.4524	0.0056
**ID**	10	-0.0218	0.9057	-0.3050	0.0896
**IL**	11	0.3060	0.0487	0.2468	0.1152
**IN**	12	0.1932	0.2203	-0.0508	0.7493
**IA**	13	0.4884	0.0019	0.4494	0.0047
**KS**	14	0.3747	0.0188	0.3742	0.0189
**KY**	15	-0.0147	0.9281	0.0643	0.6937
**LA**	16	0.2960	0.0843	0.4618	0.0052
**ME**	17	0.3483	0.1218	-0.5232	0.0149
**DC**	18	-0.0263	0.8965	-0.0530	0.7928
**MD**	18	-0.0263	0.8965	-0.0530	0.7928
**MA**	19	-0.2729	0.3064	0.3815	0.1448
**MI**	20	0.2525	0.2339	-0.0049	0.9819
**MN**	21	0.2096	0.2065	0.3587	0.0270
**MS**	22	0.1126	0.4889	0.4066	0.0092
**MO**	23	0.1041	0.5171	0.1289	0.4217
**MT**	24	0.3863	0.0290	0.4597	0.0081
**NE**	25	0.2697	0.1065	0.1558	0.3573
**NV**	26	0.0478	0.8092	-0.0285	0.8855
**NH**	27	-0.0461	0.8560	-0.0186	0.9415
**NJ**	28	0.0071	0.9745	-0.0917	0.6772
**NM**	29	-0.1588	0.4384	-0.0573	0.7809
**NY**	30	0.4117	0.0140	-0.3821	0.0235
**NC**	31	0.4492	0.0060	-0.3822	0.0214

**ND**	32	0.3367	0.0798	0.2603	0.1810
**OH**	33	0.3355	0.0343	-0.4102	0.0086
**OK**	34	0.1726	0.3069	0.2335	0.1642
**OR**	35	0.0007	0.9965	-0.1419	0.3888
**PA**	36	-0.0272	0.8712	0.0109	0.9480
**RI**	37	-0.8011	0.0168	0.6985	0.0540
**SC**	38	0.4528	0.0056	-0.4268	0.0094
**SD**	39	0.2350	0.1954	0.1028	0.5756
**TN**	40	0.3071	0.0572	-0.1613	0.3266
**TX**	41	0.3905	0.0116	0.3295	0.0354
**UT**	42	0.3147	0.0656	-0.2896	0.0916
**VT**	43	0.1588	0.4917	-0.2120	0.3561
**VA**	44	0.3589	0.0402	0.1838	0.3058
**WA**	45	0.0707	0.6774	0.2018	0.2311
**WV**	46	0.0711	0.6586	0.0390	0.8089
**WI**	47	0.5023	0.0047	-0.4558	0.0114
**WY**	48	-0.0536	0.8034	0.0890	0.6792

C :correlation coefficients; ***p*** : p-values

Table S7: Correlation coefficient between urbanland and cropland areas and DPW.

STATE	ID	Urban land		Crop land	
		C	*p*	*C*	*p*
**AL**	1	-0.3076	0.0603	0.2677	0.1042
**AZ**	2	-0.4417	0.0089	0.4477	0.0079
**AR**	3	-0.3181	0.0454	-0.0716	0.6605
**CA**	4	-0.0220	0.8941	-0.0442	0.7892
**CO**	5	-0.2712	0.1400	-0.0641	0.7320
**CT**	6	-0.6697	0.0012	0.7053	0.0005
**DE**	7	-0.2199	0.5697	0.2457	0.5239
**FL**	8	0.1676	0.3286	-0.0003	0.9988
**GA**	9	0.0859	0.6185	-0.2127	0.2130
**ID**	10	-0.3711	0.0365	-0.4851	0.0049
**IL**	11	-0.0910	0.5664	-0.0355	0.8235
**IN**	12	-0.3056	0.0491	0.3450	0.0252
**IA**	13	0.2118	0.2018	0.2238	0.1769
**KS**	14	-0.1281	0.4370	-0.0513	0.7565
**KY**	15	-0.3834	0.0146	0.0786	0.6299
**LA**	16	-0.0031	0.9861	0.3441	0.0429
**ME**	17	-0.0710	0.7597	-0.0918	0.6922
**DC**	18	-0.3640	0.0620	0.2664	0.1792
**MD**	18	-0.3640	0.0620	0.2664	0.1792
**MA**	19	-0.5457	0.0288	0.6241	0.0098
**MI**	20	-0.0305	0.8876	0.1998	0.3492
**MN**	21	-0.1158	0.4888	0.2127	0.1997
**MS**	22	-0.3816	0.0151	0.4515	0.0035
**MO**	23	-0.2846	0.0713	-0.0973	0.5451
**MT**	24	-0.0100	0.9566	0.0695	0.7054
**NE**	25	-0.2649	0.1131	-0.1841	0.2754
**NV**	26	-0.2211	0.2582	-0.2202	0.2602
**NH**	27	-0.4667	0.0509	0.4175	0.0847
**NJ**	28	-0.2528	0.2445	0.1387	0.5280
**NM**	29	-0.5527	0.0034	-0.0153	0.9410
**NY**	30	-0.0414	0.8133	0.0678	0.6987
**NC**	31	-0.0457	0.7913	0.0726	0.6741

**ND**	32	0.0716	0.7171	0.2159	0.2699
**OH**	33	-0.0260	0.8733	-0.1794	0.2679
**OK**	34	-0.1842	0.2751	-0.0461	0.7865
**OR**	35	-0.3453	0.0313	-0.0679	0.6812
**PA**	36	-0.3568	0.0279	0.3356	0.0394
**RI**	37	-0.8804	0.0039	0.7712	0.0251
**SC**	38	-0.1143	0.5067	0.1255	0.4657
**SD**	39	-0.0703	0.7024	-0.1602	0.3811
**TN**	40	-0.2421	0.1376	0.2942	0.0691
**TX**	41	-0.1076	0.5033	0.1637	0.3064
**UT**	42	-0.0692	0.6928	-0.1265	0.4690
**VT**	43	-0.1288	0.5779	0.0857	0.7118
**VA**	44	0.0251	0.8895	0.2705	0.1278
**WA**	45	-0.3326	0.0443	-0.0896	0.5979
**WV**	46	-0.2037	0.2015	0.1918	0.2297
**WI**	47	0.1741	0.3575	-0.1239	0.5142
**WY**	48	-0.4418	0.0307	-0.3235	0.1231

C :correlation coefficients; ***p*** : p-values

Table S8: Portion of flood damage variation explained by the statistical model.

State id	R^2 statistic	p-value
**1**	0.34002	0.00696
**2**	0.31805	0.02180
**3**	0.40923	0.00082
**4**	0.40914	0.00104
**5**	0.16873	0.28900
**6**	0.50099	0.02589
**7**	0.63563	0.30155
**8**	0.51863	0.00011
**9**	0.59752	0.00001
**10**	0.34908	0.01736
**11**	0.40291	0.00061
**12**	0.23428	0.03823
**13**	0.62219	0.00000
**14**	0.57651	0.00000
**15**	0.26929	0.02357
**16**	0.43490	0.00144
**17**	0.36090	0.10819
**18**	0.59095	0.00040
**19**	0.19630	0.62519
**20**	0.39146	0.04213
**21**	0.21811	0.07936
**22**	0.76273	0.00000
**23**	0.31848	0.00677
**24**	0.68087	0.00000
**25**	0.20564	0.10784
**26**	0.20784	0.23257
**27**	0.10358	0.82203
**28**	0.09261	0.76460
**29**	0.19996	0.29784
**30**	0.44142	0.00123
**31**	0.43047	0.00125
**32**	0.27698	0.10045
**33**	0.43501	0.00039

**34**	0.19025	0.13817
**35**	0.34975	0.00461
**36**	0.28587	0.02211
**37**	0.80502	0.19006
**38**	0.45649	0.00064
**39**	0.23064	0.11939
**40**	0.46201	0.00023
**41**	0.41342	0.00057
**42**	0.46709	0.00064
**43**	0.43521	0.04640
**44**	0.29808	0.03646
**45**	0.27909	0.02909
**46**	0.23471	0.04236
**47**	0.48241	0.00187
**48**	0.17714	0.42091

Figure S1: Temporal changes in flood damage, GDP and total annual runoffs during the study period.

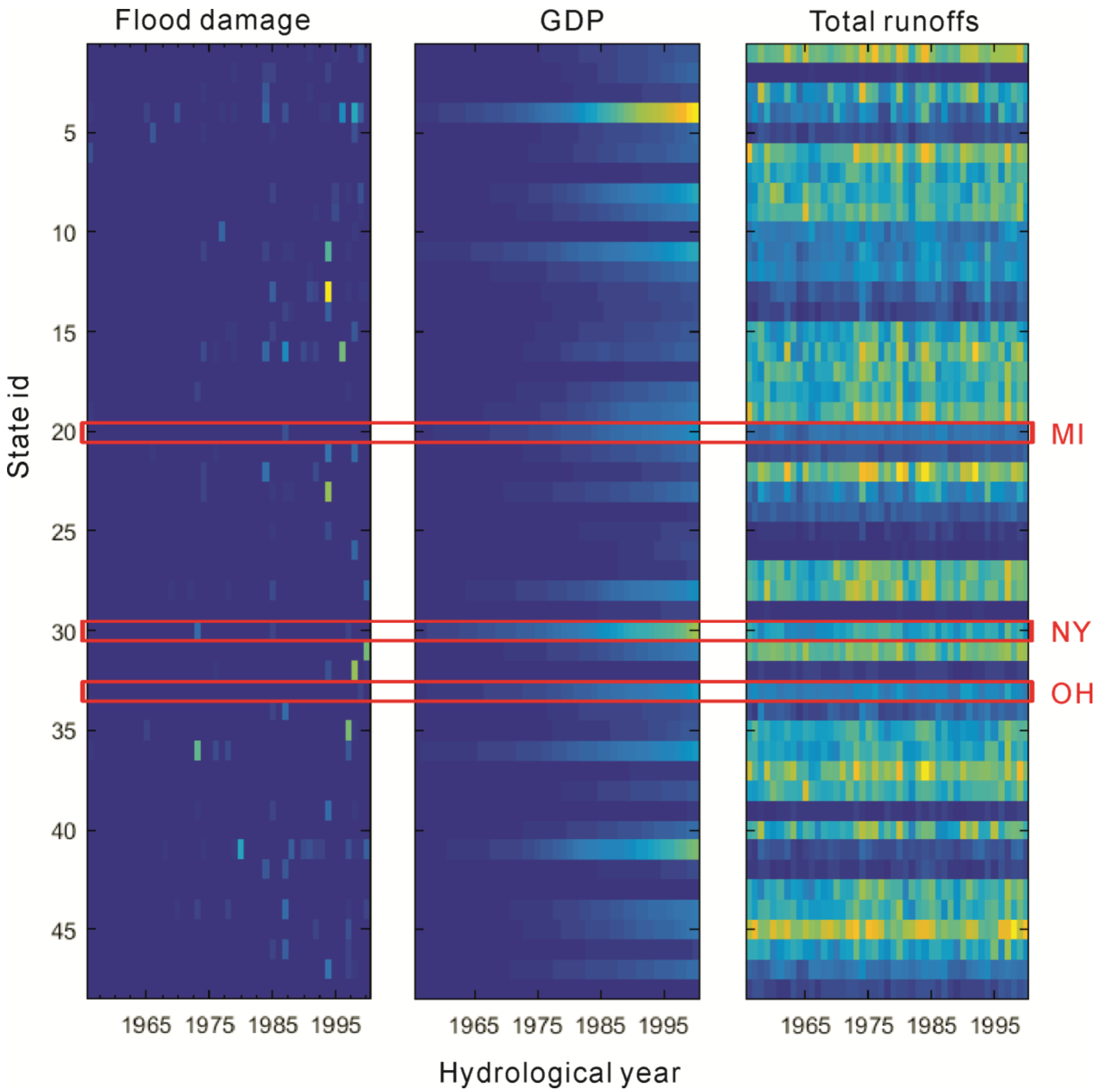

Supplement: Supplementary file 1 — Supplementary materials [file 41598_2017_5773_MOESM1_ESM.pdf]
